# Thalamic Volume Mediates Associations Between Cardiorespiratory Fitness and Memory in People With Parkinson’s

**DOI:** 10.1093/geroni/igaa057.1619

**Published:** 2020-12-16

**Authors:** Andrew Petkus, Megan Gomez, Dawn Schiehser, Vincent Filoteo, Jennifer Hui, Behnaz Jarrahi, Sarah McEwen, Giselle Petzinger

**Affiliations:** 1 USC Keck School of Medicine, Los Angeles, California, United States; 2 VA Long Beach Healthcare System, Long Beach, California, United States; 3 VA San Diego Healthcare System, San Diego, California, United States; 4 University of Southern California, LOS ANGELES, California, United States; 5 Stanford University, Stanford, California, United States; 6 Pacific Neuroscience Institute, Santa Monica, California, United States

## Abstract

Cognitive deficits occur in patients with Parkinson’s disease (PD), and cardiorespiratory fitness (CRF) is associated with both current and future cognitive decline in this disease. The underlying neurobiological factors explaining this relationship, however, are not well known. In this cross-sectional study we examined the associations between CRF and cognitive performance and whether such associations were mediated by grey matter volumes of basal ganglia structures. A total of 33 individuals with PD underwent structural magnetic resonance imaging (sMRI), CRF evaluation (VO2max), and neuropsychological assessment. Composite scores of episodic memory, executive functioning, attention, language, and visuospatial functioning were generated. Brain MRI morphological measurements was performed with the Freesurfer image analysis suite. Structural equation models were constructed to examine whether sMRI volume estimates of basal ganglia structures, specifically the thalamus and pallidum, mediated associations between VO2 max and cognitive performance while adjusting for age, education, PD disease duration, sex, and intracranial volume. Higher VO2max was associated with better episodic memory (Standardized β=0.390; p=0.009), executive functioning (Standardized β=0.263; p=0.021), and visuospatial performance (β=0.408; p=0.004). Higher VO2max was associated with larger thalamic (Standardized β=0.602; p<0.001) and pallidum (Standardized β=0.539; p<0.001) volumes. Thalamic volume significantly mediated the association between higher VO2max and better episodic memory (indirect effect=0.209) and visuospatial ability (indirect effect=0.178) performance (p<.05). The pallidum did not significantly mediate associations between VO2 max and cognitive outcomes. These results suggest the thalamus plays an important role in the association between CRF episodic memory and visuospatial functioning in individuals with PD.

